# Effects of exercise and horticultural intervention on the brain and mental health in older adults with depressive symptoms and memory problems: study protocol for a randomized controlled trial [UMIN000018547]

**DOI:** 10.1186/s13063-015-1032-3

**Published:** 2015-11-04

**Authors:** Hyuma Makizako, Kota Tsutsumimoto, Takehiko Doi, Ryo Hotta, Sho Nakakubo, Teresa Liu-Ambrose, Hiroyuki Shimada

**Affiliations:** Department of Preventive Gerontology, Center for Gerontology and Social Science, National Center for Geriatrics and Gerontology, 7-430 Morioka-cho, Obu, Aichi 474-8551 Japan; Japan Society for the Promotion of Science, Kojimachi Business Center Building, 5-3-1 Kojimachi, Chiyoda-ku, Tokyo 102-0083 Japan; Aging, Mobility, and Cognitive Neuroscience Laboratory, Djavad Mowafaghian Centre for Brain Health, University of British Columbia, 212-2177 Wesbrook Mall, Vancouver, BC V6T 1Z3 Canada

**Keywords:** Cognition, Depression, Exercise, Hippocampal volume, Horticulture

## Abstract

**Background:**

Depressive symptoms and memory problems are significant risk factors for dementia. Exercise can reduce depressive symptoms and improve cognitive function in older people. In addition, the benefits of horticultural activity on physical and mental well-being have been demonstrated in people with dementia. Although evidence of such non-pharmacological interventions is mounting, no studies have examined whether physical exercise and horticultural activity exert a positive impact on brain and mental health (e.g., depressive symptoms) in non-demented older adults at high risk of cognitive impairment and depression. Therefore, we propose a randomized controlled trial to assess the efficacy and efficiency of physical exercise and horticultural activity in improving brain and mental health in community-dwelling older adults with memory problems and depressive symptoms.

**Methods/Design:**

The 20-week randomized controlled trial will include 90 community-dwelling adults aged 65 years or older with memory problems and depressive symptoms. Participants will be randomized to one of three experiments: exercise, horticultural activity, or educational control group, using a 1:1:1 allocation ratio. The combined exercise program and horticultural activity program will consist of 20 weekly 90-minute sessions. Participants in the exercise group will practice aerobic exercise, muscle strength training, postural balance retraining, and dual-task training. The horticultural activity program will include crop-related activities, such as field cultivation, growing, and harvesting. Participants in the educational control group will attend two 90-minute educational classes during the 6-month trial period. Depressive symptoms and memory performance will be measured by the Geriatric Depression Scale-15, and the Logical Memory subtests of the Wechsler Memory Scale-Revised will be used to measure depressive symptoms and memory performance as primary outcomes, at baseline (prior to randomization), immediately following intervention (6 months from baseline), and 6 months after intervention. Hippocampal volume will be measured at baseline and immediately after intervention, using magnetic resonance imaging. Secondary outcomes will comprise cognitive function, including language, attention/executive performance, and processing speed; brain-derived neurotrophic-factor serum levels; and health-related quality of life.

**Discussion:**

This intervention study will determine the clinical importance and efficacy of physical exercise and horticultural activity as non-pharmacological interventions in community-dwelling older adults at high risk of poor brain and mental health.

**Trial registration:**

UMIN000018547; registered 7 August 2015.

## Background

Depressive symptoms constitute a significant risk factor for Alzheimer’s disease (AD) [1]. Epidemiological studies have reported an association between depressive symptoms and cognitive decline, and depressive symptoms have been shown to predict cognitive decline in old age [2–4]. Several factors affect cognitive functioning in old age, and older people with memory problems are considered to be at a higher risk of developing dementia, particularly AD, relative to those without memory problems [5, 6]. In addition, cognitive impairment is more common in older people with depression [7]. Therefore, older adults with combined memory decline and depressive symptoms may be at a higher risk of dementia, and they should be the focus of interventions designed to improve brain and mental health and address issues such as memory problems and depressive symptoms.

Exercise can improve cognitive function and reduce depressive symptoms in older people. Specifically, aerobic exercise has been shown to produce mild-to-moderate cognitive gains in healthy adults [8]. Cognitive improvement has also been observed following aerobic exercise in older adults with mild cognitive impairment (MCI) [9, 10]. However, evidence concerning the effects of aerobic exercise on memory in older adults with a higher risk of cognitive impairment is limited [11]. Some studies have shown memory improvement following exercise interventions involving physical and cognitive activities in participants with MCI [12, 13]. Interestingly, moderate-intensity physical exercise may increase hippocampal volume in cognitively healthy community-dwelling older adults [14] and those with MCI [15]. Hippocampal volume is the primary determinant of memory decline [16], and geriatric depression magnifies hippocampal atrophy and the risk of AD [17].

Exercise could also improve mood in older people. Antidepressant effects have been observed with exercise in people with mild depression [18]. A systematic review indicated that exercise was moderately more effective than a control intervention in reducing symptoms of depression, but analysis of methodologically robust trials showed a smaller effect in favor of exercise [19]. Results of another review were consistent with the suggestion that, for older people who present with clinically meaningful symptoms of depression, prescribing structured exercise with mixed elements of endurance and strength training tailored to individual ability is likely to reduce the severity of depression [20].

Another non-pharmacological intervention strategy for reducing depression is horticultural activity, which is expected to increase social and behavioral activation and mental well-being, and moderate levels of physical activity in a nature-based environment [21]. In addition, intervention studies, randomized controlled trials (RCTs), and pre–post design studies have been conducted to examine the effects of horticultural activity on physical and mental well-being in people with dementia, and the results showed reductions in levels of agitation [22]. However, the quantitative studies were of poor quality with respect to sample size and study design. In addition, there have been no well-designed intervention studies conducted to examine the effects of horticultural activity on brain and mental health (e.g., depressive symptoms, cognitive function, and brain volume) in non-demented adults with a higher risk of dementia.

We hypothesize that physical exercise and horticultural activity may exert a positive impact on brain (e.g., cognitive function and brain volume in the hippocampus) and mental health (e.g., depressive symptoms) in older adults with a higher risk of cognitive impairment and depression. However, this hypothesis has not been tested. Therefore, we propose a 20-week RCT involving community-dwelling adults aged 65 years or older with mild memory problems and depressive symptoms. Further, we aim to explore the relative importance of hippocampal volume changes with respect to improvements in cognitive function and mood. Given the immense health and financial burdens imposed by dementia and depression, the results of our proposed RCT could exert a significant impact on the health of Japanese seniors and the country’s long-term care system.

## Methods/Design

### Study design

**Fig. 1:**
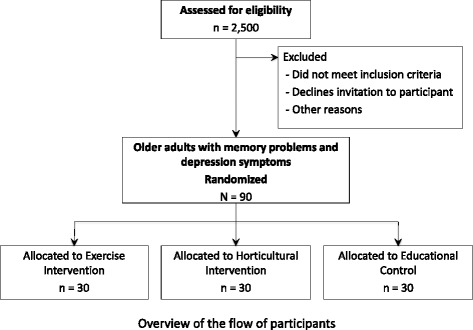
Overview of the flow of participants

The proposed study is a randomized single-blind controlled community-based trial with a parallel design and a 1:1:1 allocation ratio. The study design is shown in Fig. 1. We will recruit 90 community-dwelling adults aged 65 years or older with memory problems and depressive symptoms. Informed consent will be obtained from all participants prior to their inclusion in the study. The study protocol was approved by the Ethics Committee of the National Center for Geriatrics and Gerontology (#839).

### Setting

This study will be conducted in the Japanese community in Obu city, a suburb of Nagoya, Japan. Obu city has a population of 88,550, of whom 17,354 (19.6 %) are aged 65 or older (in April 2014). We have been conducting an observational study, including face-to-face interviews and measures of physical and cognitive function, in this community since 2011 [23]. Pre-intervention and post-intervention participant screening and data collection will be performed at the community center. Magnetic resonance imaging (MRI) scanning will be conducted at the Research Institute at the National Center for Geriatrics and Gerontology.

### Study population

The study population will include community-dwelling older adults with mild memory decline and depressive symptoms. Participants will be required to meet the following inclusion criteria: (1) independent adults aged 65 years or older living within the community; (2) memory problems (subjective memory complaints or objective mild memory decline indicated via an age-adjusted word list memory score at least 1.0 SD below the reference threshold); and (3) presence of depressive symptoms (Geriatric Depression Scale-15 [GDS-15] score of ≥5) [24]. Participants will be excluded if they meet the following exclusion criteria: (1) support or care certified by the Japanese public long-term care insurance system; (2) dementia diagnosis or Mini-Mental State Examination score of ≤18; (3) a history of major psychiatric illness (e.g., bipolar disorder) or other serious neurological or musculoskeletal diagnoses; (4) disability in basic activities of daily living; (5) incapable of undertaking cognitive performance tests; (6) physical exercise contraindication; and (7) use of walking aids in daily life. Participants will also be excluded if they cannot sign the informed consent form.

### Sample size

The required sample size for this study was calculated using predictions of 6-month changes in GDS-15 results. Based on the results of our previous work, conducted in the same town, we predict that a change of 1.5 GDS-15 points would indicate differences and assume that the SDs for the GSD-15 would be 2.5 in our sample. Assuming a non-consent and dropout rate of 20–30 %, 30 participants are required per group. The study design necessitates power of at least 80 % and an α level of <.05 to assess the effects of the interventions.

### Randomization

Participants will be randomly assigned (1:1:1) to an exercise intervention (EI), a horticultural intervention (HI), or an educational (control) group upon completion of baseline assessments. The randomization sequence will be computer generated [25, 26]. A researcher who is unaware of the aims of the study will perform the randomization procedure.

### Planned trial interventions

#### Exercise intervention group

The combined exercise program will involve 20 weekly 90-minute sessions involving physical and cognitive activities. Approximately 10–15 individuals will participate in each class at a fitness facility. Trained instructors will conduct the exercise sessions, which will involve aerobic exercise, muscle strength training, postural balance retraining, and dual-task training. Each session will begin with a 10-minute warm-up period and stretching exercises followed by 20 minutes of muscle strength exercise and postural balance retraining. The exercises will be conducted under multitask conditions that will include physical and cognitive tasks; we have called this combination training “cognicize.” For example, participants play word games while engaging in stepping exercises. The participants will also undertake daily home-based exercises and walking, which will require self-monitoring using a booklet and pedometer. To improve health behavior, physiotherapists will deliver lectures to inform the participants about cognitive health, exercise methods, the risks associated with dementia, effects of exercise on dementia, and ways in which to self-monitor regular physical activities.

#### Horticultural intervention group

The HI program will involve 20 weekly 90-minute sessions involving nature-based activities. The program will include crop-related activities such as cultivating a field, growing, and harvesting. Individuals in this group will engage in gardening activities including group planting (known as *Yoseue style bonsai*), which involves the combination of different plant varieties or shapes, and planting flowers in the public garden. The vegetable experts will lecture the participants with nutritional information and recipes for the field crops that will be grown in the program.

#### Educational control group

Participants in the educational control group will attend two 90-minute education classes during the 6-month trial period. The classes will include topics that experts consider less likely to influence study outcomes (e.g., effective remedies for crime or traffic accidents in older people).

### Outcomes

Outcomes will be measured at the community center by research assistants who are blinded to the randomization status. Participants will be required to take part in baseline data collection lasting approximately 2 hours. Subsequent data collection will occur immediately following the intervention (6 months after baseline) and 6 months after intervention for all outcomes. All outcome measures will be assessed by independent examiners who are unaware of group allocation.

#### Primary outcomes

Primary outcomes will include a reduction in depressive symptoms, based on changes in GDS-15 scores, and an improvement in memory performance, assessed via word list memory [27] and the Logical Memory subtests of the Wechsler Memory Scale-Revised (WMS-R) [28]. The GDS-15 consists of 15 items. A score of 5 or higher will be used to identify clinical depressive symptoms [29, 30].

The word list memory tasks will involve immediate recognition and delayed recall of a 10-word target list [27]. Participants are instructed to memorize 10 words, which are presented on a tablet personal computer. Each of the 10 target words is presented for 2 seconds. A total of 30 words, including 10 target and 20 distracter words, is then presented, and participants are asked to choose the 10 target words immediately (Word-List Memory Task I); this is repeated for three trials. The average number of correct answers is calculated to produce a score within a range of 0–10. In addition, participants are instructed to recall (and record in writing) the 10 target words after approximately 20 minutes (Word-List Memory Task II). The total number of target words recalled is then calculated.

In the WMS-R Logical Memory subtests, two short stories (A and B) are read aloud to the participant, who is instructed to recall the details of the stories immediately (Logical Memory Task I) and after 30 minutes (Logical Memory Task II) [28]. Total scores are calculated (i.e., sum of scores for stories A and B) for WMS-R Logical Memory Tasks I and II.

#### Secondary outcomes

Secondary outcomes will include whole-brain and hippocampal volume, evaluated using MRI; cognitive function, assessed using verbal fluency tests (VFTs) [31] and tablet versions of the trail-making test (TMT) [27] and symbol digit substitution test (SDST) [27]; brain-derived neurotrophic factor (BDNF) serum levels; and health-related quality of life (QOL).

Whole-brain and hippocampal volume will be determined using a 3-T system (TM Trio, Siemens, Germany). Three-dimensional volumetric acquisition of a T1-weighted gradient echo sequence will be performed to produce a gapless series of thin sagittal sections using a magnetization preparation rapid-acquisition gradient-echo sequence (inversion time [TI] 800 ms; repetition time [TR] 1800 ms; echo time [TE] 1.98 ms; and 1.1 mm slice thickness). Axial T2-weighted spin-echo images (TR 4200 ms; TE 89.0 ms; and 5.0 mm slice thickness) and axial fluid-attenuated inversion-recovery images (TI 2500 ms; TR 9000 ms; TE 100 ms; 5 mm slice thickness) will then be obtained for diagnosis.

Verbal fluency will be assessed according to the number of words generated across 60-second trials [32]. Both the letter VFT (which assesses phonemic verbal fluency) and the animal naming test (which assesses semantic verbal fluency) will be performed. In the letter VFT, participants will be instructed to retrieve as many words (excluding proper names) as possible within a 60-second period, beginning with the Japanese syllabic characters (hiragana) “Shi,” “I,” and “Re” [33]. The total number of words generated for all three letters will be used as a measure of performance. In the animal naming test, participants will be instructed to generate a list of animal names within 60 seconds [31]. The tablet version of the TMT, consisting of parts A and B, will be used to determine attention and executive function [27]. In the tablet version of the TMT-A, participants are required to touch target numbers (1–15), which are presented randomly on the panel, in consecutive order as rapidly as possible. In the tablet version of the TMT-B, participants touch target numbers or letters, alternating between consecutive numbers and letters (Japanese Kana characters). We will record the time (in seconds) taken to complete each task; a shorter time represents superior performance. In the tablet version of the SDST, nine pairs of numbers and symbols are presented at the top of the display, and a target symbol is shown in the center of the display. Participants then choose a number that corresponds to a target symbol, which is presented at the bottom of the display, as rapidly as possible. The number of correct numbers chosen within 90 seconds constitutes the score. One point is awarded for each number that is chosen correctly within the time limit. The tablet versions of the TMT and SDST have demonstrated reliability and validity in a sample of community-dwelling older adults [27].

Serum BDNF levels will be measured using the Quantikine Human Kit (R & D Systems, Inc. Minneapolis, MN, USA) and used as a biomarker [12].

Health-related QOL will be assessed using the Short-Form Health Survey-12, a standardized instrument with established psychometric validity, which measures eight health domains: physical functioning, role limitations due to physical health, bodily pain, general health, vitality (energy/fatigue), social functioning, role limitations due to emotional health, and mental health (psychological distress and wellbeing) [34].

#### Other outcomes

Other outcomes will include physical performance tests such as handgrip strength, walking speed, and the two-minute walking test [35]; social network, assessed using the abbreviated version of the Lubben Social Network Scale (LSNS-6) [36]; Life-Space Assessment (LSA) [37]; subjective sleep quality, assessed using the Pittsburgh Sleep Quality Index [38]; and daily physical activity level, assessed using a triaxial accelerometer [39]. We will monitor adherence to the intervention programs and record adverse events.

Physical performance tests will include handgrip strength to measure muscle strength, normal and maximum walking speed to measure general gait ability, and the two-minute walking test to assess exercise tolerance. Handgrip strength and walking speed are simple, easy to implement in community settings, and strong predictors of health outcomes [40]. In the two-minute walking test, participants will be asked to walk, covering as much ground as possible in a demarcated area within 2 minutes. The distance walked within 2 minutes will be measured [35].

Social network will be measured using the LSNS-6, which is a six-item scale (simple sum scoring with a range of 0–30) consisting of three items that evaluate kinship ties and three items that evaluate non-kin ties [36]. Life-space mobility will be measured via the LSA, which is used to obtain a score based on the reported distance travelled during the 4 weeks preceding assessment. Scores range from 0 (“totally roombound”) to 120 (“travelled out of town every day without assistance”), with lower scores reflecting lower life-space mobility [37, 41]. Sleep quality will be evaluated using the Pittsburgh Sleep Quality Index, which consists of 19 items that produce a global sleep quality score and scores for the following seven components: sleep quality, latency, duration, disturbance, habitual sleep efficiency, use of soporific medication, and daytime dysfunction. The sum of these seven component scores yields one global score for subjective sleep quality (range 0–21), with higher scores reflecting poorer subjective sleep quality [42]. Daily physical activity level, including duration of light, moderate, and total physical activity, and the numbers of steps taken during the 2-week periods subsequent to pre-intervention and post-intervention assessments, will be measured using a triaxial accelerometer [39].

### Statistical analyses

Statistical analyses will be conducted to assess the effects of interventions according to the intention-to-treat principle, which states that data for all individuals should be analyzed according to their group allocation, regardless of compliance. Data will be entered according to a multiple imputation method. Between-group differences in primary outcomes subsequent to intervention will be compared using multiple linear regression analysis. Baseline scores, experimental groups, and characteristics (e.g., age, sex, diagnoses, and medical conditions) will be included in the models as covariates. If the effects of the intervention are significant, two planned simple contrasts will be performed to assess differences between the EI and control groups and the HI and control groups. Contrasts will also be performed within each intervention group to determine whether intervention benefits are observed for the primary outcomes. The analysis performed for the primary outcomes will also be performed for the secondary and other outcome measures.

## Discussion

This RCT will determine the clinical importance and efficacy of non-pharmacological interventions in community-dwelling older adults at a higher risk of poor brain and mental health, with MRI data and biomarkers used to assess the biological mechanisms of action in multidimensional exercise and horticultural activity.

Improved understanding of the primary mechanisms underlying exercise and horticultural activity would increase our capacity to refine and develop non-pharmacological interventions for dementia and depression prevention in the aging population. If interactions between memory improvement, brain atrophy control, and depression reduction are clarified, these results could make a major contribution to knowledge in this field.

One of strengths of our study is that the findings could easily be translated into evidence-based intervention strategies that both professionals and the lay public could use to engage older adults in exercise and horticultural activity. If this study provides evidence of a potential treatment that could increase brain health, including improvements in cognitive function and mental health and reductions in brain atrophy, our findings could be used immediately as non-pharmacological interventions for dementia and depression prevention in the community.

## Trial status

At the time of manuscript submission, we have obtained ethical approval, registered the trial, and successfully recruited 29 participants, and the status of the trial is open for enrollment.

## Abbreviations

AD: Alzheimer’s disease
BDNF: Brain-derived neurotrophic factor
EI: Exercise intervention
GDS-15: Geriatric Depression Scale-15
HI: Horticultural intervention
LSA: Life-Space Assessment
LSNS-6: Lubben Social Network Scale
MCI: Mild cognitive impairment
MRI: Magnetic resonance imaging
QOL: health-related quality of life
RCT: Randomized controlled trial
SDST: Symbol Digit Substitution Test
TMT: Trail-Making Test
VFT: Verbal Fluency Test
WMS-R: Wechsler Memory Scale-Revised
